# Rlip Reduction Induces Oxidative Stress and Mitochondrial Dysfunction in Mutant Tau-Expressed Immortalized Hippocampal Neurons: Mechanistic Insights

**DOI:** 10.3390/cells12121646

**Published:** 2023-06-16

**Authors:** P. Hemachandra Reddy, Sudhir Kshirsagar, Chhanda Bose, Jangampalli Adi Pradeepkiran, Ashly Hindle, Sharda P. Singh, Arubala P. Reddy, Javaria Baig

**Affiliations:** 1 Department of Internal Medicine, Texas Tech University Health Sciences Center, Lubbock, TX 79430, USA; sudhir.kshirsagar@ttuhsc.edu (S.K.); chhanda.bose@ttuhsc.edu (C.B.); pradeep.jangampalli@ttuhsc.edu (J.A.P.); ashly.hindle@ttuhsc.edu (A.H.); sharda.singh@ttuhsc.edu (S.P.S.); jbaig@ttuhsc.edu (J.B.); 2 Nutritional Sciences Department, College of Human Sciences, Texas Tech University, Lubbock, TX 79409, USA; arubala.reddy@ttu.edu; 3 Neurology, Departments of School of Medicine, Texas Tech University Health Sciences Center, Lubbock, TX 79430, USA; 4 Public Health Department of Graduate School of Biomedical Sciences, Texas Tech University Health Sciences Center, Lubbock, TX 79430, USA; 5 Department of Speech, Language and Hearing Sciences, School Health Professions, Texas Tech University Health Sciences Center, Lubbock, TX 79430, USA; 6 Department of Pharmacology and Neuroscience, Texas Tech University Health Sciences Center, Lubbock, TX 79430, USA

**Keywords:** mutant Tau, Rlip deficiency, mitochondrial function, phosphorylated tau, oxygen consumption rate, mitophagy

## Abstract

RalBP1 (Rlip) is a stress-activated protein that is believed to play a large role in aging and neurodegenerative diseases such as Alzheimer’s disease (AD) and other tauopathies. The purpose of our study was to understand the role of Rlip in mutant Tau-expressed immortalized hippocampal HT22 cells. In the current study, we used mutant Tau (mTau)-expressed HT22 neurons and HT22 cells transfected with Rlip-cDNA and/or silenced RNA, and studied the cell survival, mitochondrial respiration, mitochondrial function, immunoblotting, and immunofluorescence analysis of synaptic and mitophagy proteins and the colocalization of Rlip and mTau proteins. We found Rlip protein levels were reduced in mTau-HT22 cells, Rlip silenced HT22 cells, and mTau + Rlip RNA silenced HT22 cells; on the other hand, increased Rlip levels were observed in Rlip cDNA transfected HT22 cells. We found cell survival was decreased in mTau-HT22 cells and RNA-silenced HT22 cells. However, cell survival was increased in Rlip-overexpressed mTau-HT22 cells. A significantly reduced oxygen consumption rate (OCR) was found in mTau-HT22 cells and in RNA-silenced Rlip-HT22 cells, with an even greater reduction in mTau-HT22 + Rlip RNA-silenced HT22 cells. A significantly increased OCR was found in Rlip-overexpressed HT22 cells and in all groups of cells that overexpress Rlip cDNA. Mitochondrial function was defective in mTau-HT22 cells, RNA silenced Rlip in HT22 cells, and was further defective in mTau-HT22 + Rlip RNA-silenced HT22 cells; however, it was rescued in Rlip overexpressed in all groups of HT22 cells. Synaptic and mitophagy proteins were decreased in mTau-HT22 cells, and further reductions were found in RNA-silenced mTau-HT22 cells. However, these were increased in mTau + Rlip-overexpressed HT22 cells. An increased number of mitochondria and decreased mitochondrial length were found in mTau-HT22 cells. These were rescued in Rlip-overexpressed mTau-HT22 cells. These observations strongly suggest that Rlip deficiency causes oxidative stress/mitochondrial dysfunction and Rlip overexpression reverses these defects. Overall, our findings revealed that Rlip is a promising new target for aging, AD, and other tauopathies/neurological diseases.

## 1. Introduction

Alzheimer’s disease (AD) is the most common form of dementia and it occurs in both early-onset familial and late-onset sporadic forms [1]. Age-dependent increased oxidative stress and mitochondrial abnormalities as well as genetic polymorphisms are involved in late-onset AD [2]. AD is associated with the accumulation of amyloid-beta (Aβ) and phosphorylated-tau (p-Tau), in addition to hormonal imbalance, synaptic damage, mitochondrial abnormalities, microRNA deregulation, and increased glia and astrocytes [3,4,5,6,7,8,9,10,11,12,13,14,15].

Tau is a microtubule-associated protein that is abundantly present in the axons of neurons, where it stabilizes microtubules and allows cargos and other organelles to move to nerve terminals from axons [16]. Overexpressed Tau and/or phosphorylated Tau is largely involved in the impairment of the axonal transport of mitochondria and other cellular organelles, causing synapse starvation, the depletion of ATP, and neuronal damage [17,18,19,20,21]. Genetic mutations in the Tau gene are not directly involved in familial AD [16]. However, tau hyperphosphorylation and the formation/accumulation of neurofibrillary tangles (NFT) are associated with late-onset AD via increased oxidative stress and mitochondrial dysfunction [16]. In addition, the hyperphosphorylation of tau and NFTs is also involved with several neurodegenerative diseases, such as amyotrophic lateral sclerosis, argyrophilic grain dementia, corticobasal degeneration, Creutzfeldt–Jakob disease, dementia pugilistica, diffuse NFTs with calcification, Down’s syndrome, frontotemporal dementia, Gerstmann–Straussler–Scheinker disease, Hallervorden–Spatz disease, myotonic dystrophy, Niemann–Pick disease type C, non-Guamanian motor neuron disease, Pick’s disease, postencephalitic parkinsonism, prion protein cerebral angiopathy, progressive cortical gliosis, progressive supranuclear palsy, and subacute sclerosing panencephalitis [8,16,17,18,19,20]. However, the precise role of tau hyperphosphorylation and genetic polymorphisms in the genome in the progression and pathogenesis of AD, frontotemporal dementia, and other tauopathies is not completely understood.

RalBP1 (Rlip) is a stress-activated protein that plays a crucial role in oxidative stress and mitochondrial dysfunction, and its involvement with AD and other neurodegenerative diseases is still not completely understood [22,23,24,25,26,27]. The human *RALBP1* gene encodes the 76 kDa stress-responsive, antiapoptotic ATPase enzyme RLIP76. Rlip couples clathrin-dependent endocytosis with the efflux of glutathionylated electrophilic toxins [22,23,24,25,26,27]. Rlip is transcriptionally regulated by CREB-binding protein that is important for synaptic plasticity, learning/memory, long-term potentiation, and stress protection in neurons [27].

To determine the role of Rlip, we recently studied heterozygous RLIP knockout (Rlip^+/−^) mice for phenotypic behavioral changes, oxidative stress, mitochondrial function, mitochondrial biogenesis, synaptic, and mitophagy/autophagy proteins [28]. Rlip heterozygote knockout (Rlip^+/−^) mice showed multiple phenotypic behavioral impairments, including reduced locomotor activities, motor coordination impairments, spatial learning, and memory deficits; increased mitochondrial fragmentation; and reduced mitochondrial length. Increased free radicals, lipid peroxidation, and defective mitochondrial changes were observed in Rlip^+/−^ mice. However, the role of phosphorylated tau in Rlip reduction and/or overexpression in AD progression and pathogenesis is not clear yet.

The hippocampus is an important organ of the brain in humans and other mammals. The hippocampus is involved in multiple functions, including the consolidation of information from short-term memory to long-term memory, and in spatial memory that enables navigation [29,30]. The hippocampal structure and function can be studied using genes/proteins that are expressed in hippocampal neurons in rodent models of AD and autopsied brains from healthy subjects and AD patients. Furthermore, hippocampal tissues or hippocampal cells are the best sources to study learning and memory functions, particularly normal tau and/or phosphorylated tau. Several groups have worked on immortalized mouse hippocampal neurons, referred to as HT22 cells. These cells were originally subcloned from the hippocampal cell line (HT-4) [31]. The parental HT-4 cell line was derived from the immortalization of mouse hippocampal tissues with a temperature-sensitive SV40 T-antigen [32].

**Figure 1:**
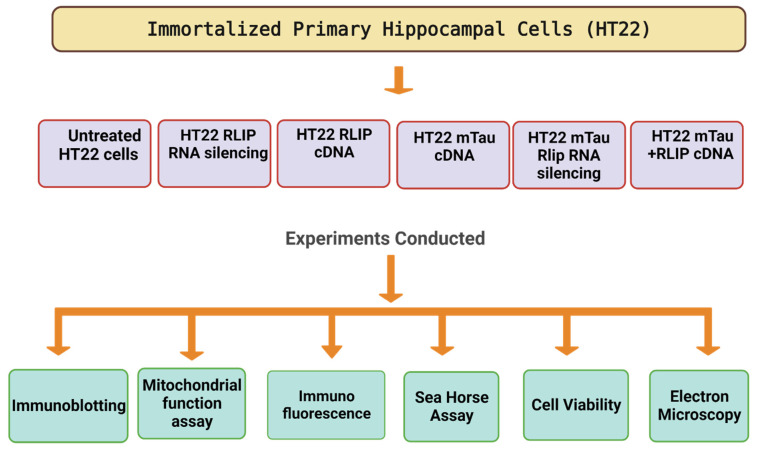
The experimental approach of HT22 cells. HT22 cells transfected with mutant Tau, Rlip RNA silencing, Rlip cDNA, and mitochondrial parameters studied.

The purpose of our article is to understand the role of RLIP in immortalized mouse hippocampal (HT22) cells that express mutant Tau. In the current study, we transfected HT22 neurons with (1) Rlip silenced (using RNA interference strategy), (2) Rlip overexpressed (using Rlip-cDNA transfected), (3) mutant Tau-cDNA transfected, (4) cotransfected both mutant Tau-cDNA transfected + Rlip RNA silencing, (5) mutant Tau-cDNA transfected + Rlip RNA silenced in comparison to HT22 cells (Figure 1).

## 2. Material and Methods

*Chemicals and reagents.* HT22 cells were kindly gifted to us by David Schubert. Dulbecco’s Modified Eagle Medium (DMEM), penicillin/streptomycin, fetal bovine serum, and Trypsin-EDTA were purchased from GIBCO (Gaithersburg, MD, USA).

### 2.1. Mutant Tau cDNA Constructs

A mutant Tau cDNA clone (P301L) was purchased from Addgene—https://www.addgene.org. The expression of P301L mutations was verified and further subcloned into a mammalian expression vector. Then, mutant Tau cDNA clone (P301L) and RALBP1 antisense locked nucleic acid (Rlip-LNA) Exiqon GapmeRs (Qiagen, Hilden, Germany) were transfected together with Rlip siRNA or Rlip cDNA into HT22 cells using lipofectamine 3000 with P3000 for 24 h; then, cells were harvested, and a pellet was collected to extract the proteins for further experiments.

### 2.2. Tissue Culture Work

The HT22 cells were grown for 2–3 days in a medium (1:1 mixture of DMEM and OptiMEM, 10% FBS, plus penicillin and streptomycin (Invitrogen, Carlsbad, CA, USA) until the cells were 60–70% confluent. We performed 6 independent cell cultures and transfections with mutant Tau cDNA treatments for all experiments (1) HT22 cells (control), (2) SiRlip (Rlip silencing), (3) Rlip-cDNA (Rlip overexpression), (4) mTau (mutant Tau transfected HT22), (5) mTau + SiRlip, and (6) mTau+Rlip-cDNA (mTau HT22 cells+Rlip-cDNA).

### 2.3. mTau Overexpression and Rlip Depletion by siRNA

5 × 10^5^ cells were seeded in 6-well plates; for chamber slides, 3 × 10^4^ cells were plated in DMEM containing 2% FBS. After the cells were attached, the medium was removed, and the cells were transfected with mTau (8 µg/mL) and Rlip-siRNA together. Rlip was depleted via transfection using Ralbp1 Mouse siRNA Oligo Duplex (Locus ID 19765) cat# SR418664, (Origene Technology Inc. MD, USA) Trilencer-27 Universal Scrambled Negative Control siRNA Duplex (cat# SR30004) suspended in RNAse-free siRNA Duplex Resuspension Buffer (cat#SR30005). Lipofectamine complexes were prepared as follows: P3000 with mTau cDNA and Rlip-siRNA or scrambled control (10 nM final concentration) and Lipofectamine 3000 reagent were separately prediluted in 500 μL of Opti-MEM Medium according to the manufacturer’s protocol, then mixed gently and incubated at room temperature for 10 min, then added to plates. Rlip siRNA or scrambled control and Lipofectamine complex were added to the cells. Growth media supplemented with 2% FBS without antibiotics were added to the cells and incubated for 24 h in a 5% CO_2_ humidified incubator at 37 °C. After 24 h, cells were trypsinized and centrifuged at 500× *g* for 5 min. Cell pellets were used for subsequent experiments as needed.

### 2.4. Cell Survival Assay

An Annexin V assay was performed to measure apoptosis using a Cellometer K2 CBA Image Cytometry System (Nexcelom Bioscience LLC, Lawrence, MA, USA) per the manufacturer’s protocol. Briefly, Annexin V-FITC and propidium iodide (PI) staining solutions were used to fluorescently label cells in order to identify apoptotic and necrotic cells. HT22 cells were seeded overnight in complete medium and transfected the next day. After 24 h, cells were trypsinized and pelleted via centrifugation. Then, the cell pellets were resuspended in 40 μL of Annexin V binding buffer. Next, 5 µL each of Annexin V–FITC reagent and PI was added. The mixture was gently mixed by pipetting and incubated at room temperature in the dark for 15 min. Then, the mixture was washed by adding 250 μL of PBS and pelleted via centrifugation. The cells were resuspended in 50 μL of Annexin V binding buffer and analyzed for a fluorescence signal using a K2 Cellometer.

### 2.5. Mitochondrial Respiration Using Seahorse XFe96 Extracellular Flux Analyzer

Mitochondrial respiration was measured using a Seahorse XFe96 extracellular flux analyzer. HT22 cells were seeded in a tissue culture dish and grown overnight. The next day, cells were transfected using Lipofectamine 3000 with P3000. Cells were then trypsinized, counted, and replated in Seahorse Bioanalyzer with 96-well plates at 10,000 cells per well in 80 μL of DMEM complete medium (values are normalized by the number of cells (10,000 cells) per well), but for four blank wells for background correction, located at positions A1, A12, H1, and H12, only 80 μL of growth medium was added. Then, the plate was kept in a cell culture hood for 1 h at room temperature to allow cells to promote uniform adherence on the well bottoms and decrease edge effects. Then, the cells were incubated overnight in a 37 °C humidified incubator. The sensory cartridge and utility plate were detached, and the sensory cartridge was placed upside down on the bench beside the utility plate. Then, 200 µL of Seahorse XF Calibrant was added to all wells of the utility plate, and the sensory cartridge was softly lowered back onto the utility plate to avoid creating bubbles. The sensory cartridge was incubated overnight in a 37 °C incubator without CO_2_. The following morning, 60 μL of medium was removed from each XF96 cell culture microplate well, leaving 20 μL of medium remaining in each well. Next, 180 μL of freshly prepared assay medium (1 mL of 200 mM glutamine, 1 mL of 100 mM pyruvate solution, and 0.1 g of D-glucose in 98 mL XF base medium) was added to each well, and the plate was placed for 1 h in a 37 °C incubator without CO_2_. During this time, the stock solutions of oligomycin, FCCP, and rotenone/antimycin A were prepared per the manufacturer’s instructions. After the 1 h incubation, 20 μL of 1.5 μM oligomycin, 22 μL of 1 μM FCCP, and 25 μL of 0.5 μM rotenone/antimycin A were loaded in port A, B, and C of the sensory cartridge, respectively. After incubation, the utility plate and sensory cartridge were placed in the Seahorse instrument for calibration. Then, the utility plate was exchanged with the XF96 cell culture microplate to obtain the OCR measurements, which took 1 h and 24 min. After the measurements were complete, the results were automatically analyzed using the wave software, and the data were exported as Excel or GraphPad Prism files. The results are presented as mean ± SEM from six to eight replicate wells.

### 2.6. Mitochondrial Function Analysis

Mitochondrial functional analysis for lipid peroxidation, H202, and ATP levels in control and experimental cells was measured by ELISA.

**Lipid peroxidation assay (4-hydroxy-nonenol (4-HNE) levels.** Using a lipid peroxidation assay ELISA kit (cat#-238538, Abcam, Cambridge, MA, USA) 4-HNE, a biomarker of oxidative stress was measured by the HNE-protein adducts present in cells, according to the manufacturer’s instructions. In brief, 50 µg of total cell lysates was used to measure the adduct. 4-HNE protein adducts present in the sample or standard were probed with the primary 4-HNE antibody, followed by a horseradish peroxidase (HRP)-conjugated secondary antibody. Optical density was measured at 450 nm to quantify the level of HNE. The 4-HNE protein adduct content in the unknown samples was determined by comparing it with the standard curve that was prepared from predetermined HNE-BSA standards.

**H_2_O_2_ production** was measured using an H_2_O_2_ assay kit (cat#ab272537 Abcam, USA). Briefly, the reaction mixture containing reagents A and B was added to cell lysates. Then, the mixture was incubated at room temperature for 30 min. After incubation, the samples were analyzed with a microplate reader at an absorbance of 585 nm. Using a standard curve equation expressed in µmol/µg of the protein, H_2_O_2_ production was determined.

**ATP levels** were measured using an ATP assay kit (cat#83355, Abcam, Boston, MA, USA). As per the instructions, ATP levels were measured in the cell’s mitochondria. In brief, cell lysates were mixed with reaction mix including ATP reaction buffer, ATP probe, ATP converter, and developer mix. Then, the reaction was incubated at room temperature for 30 min and the absorbance was measured at 570 nm. The concentration of ATP levels in the samples was determined using a known concentration of ATP standard curve equation.

### 2.7. Western Blot Analysis

Western blot analysis was performed using protein lysates prepared from mTau HT22 cells transfected with Rlip siRNA and Rlip cDNA for 24 h. We used beta-actin as an internal control to normalize Rlip, P-Tau, synaptic, and mitophagy protein levels. Details of antibody dilutions are given in Table 1. After treatment, the cells were lysed in 50 μL of cold RIPA lysis buffer (Millipore Sigma Aldrich Corporation, Burlington, MA, USA) (20–188) for 45 min on ice (vortexed every 15 min interval) and centrifuged at 12,000 g for 12 min. After centrifugation, the supernatant was collected and protein concentration was measured. Then, 20 μg of proteins was loaded and separated using SDS-PAGE gels (10%) electrophoretically and transferred to a polyvinylidene difluoride membrane (Bio-Rad Incorporation, 10026933). Blocking was performed by adding 5% BSA for 30 min at room temperature on a shaker. After washing three times, the primary antibody was added to the membranes overnight on a shaker at 4 °C. Membranes were washed 3 times with TBST and incubated with HRP (horseradish peroxidase)-labeled secondary antibodies for 1 h at room temperature. Proteins were detected with chemiluminescence reagents (ECL, Thermo Scientific, Waltham, MA, USA, Cat#WA317048), and the band exposures were kept within the linear range.

### 2.8. Immunofluorescence Analysis

25 × 103 cells/chamber were seeded in 4-well BD chamber slides (Fisher Scientific) in DMEM containing 2% FBS. After mTau expression and Rlip siRNA transfection, as described in the Methods section, immunofluorescence staining was performed to check the expression of Tau and Rlip (Table 2). In brief: Cells were washed with 1xPBS and fixed with 4% neutral buffer formaldehyde for 15 min at room temperature. Slides were washed with 1x PBS three times for 5 min each. Cells were incubated with 0.1% triton x-100 and 5% goat serum for 30 min at room temperature. After 3 washes with 1XPBS, anti-Rlip or phosphoTau antibodies in 1% BSA and 0.1% triton x-100 in 1x PBS were added to the cells and incubated at 4 °C overnight. After 3 washes with 1xPBS for 5 min each, goat anti-rabbit IgG Alexa Fluor 488 and Texas-Red-conjugated anti-mouse IgG were diluted in 5% goat serum in 1x PBS and added to the cells. After incubation for 1 h at room temperature, the cells were washed 5 times with 1x PBS for 5 min each and mounted with Vectashield mounting media (Vector Laboratories, Burlingame, CA, USA), containing DAPI for counterstaining the nuclei. IgG only was used as a negative control. Slides were imaged with a Leica fluorescence microscope with a Leica DMC4500 camera (Leica, Wetzlar, Germany) at 400× magnification. Fluorescence intensity was quantified and analyzed using Image J software, version 1.46.

### 2.9. Transmission Electron Microscopy

Transmission electron microscopy was performed to determine the mitochondrial number and size in all the control and experimental groups of cells: HT22 cells (control), SiRlip, Rlip-cDNA, mTau, mTau+SiRlip, and mTau+Rlip-cDNA. Cells were fixed in 100 μm of sodium cacodylate (pH 7.2), 2.5% glutaraldehyde, 1.6% paraformaldehyde, 0.064% picric acid, and 0.1% ruthenium red. They were gently washed and postfixed for 1 h in 1% osmium tetroxide plus 8% potassium ferricyanide in 100 mm sodium cacodylate, pH 7.2. After thoroughly rinsing in water, the HT22 cells were dehydrated, infiltrated overnight in 1:1 acetone: Epon 812, and infiltrated for 1 h with 100% Epon 812 resin. Ultrathin sections were cut, stained with uranyl acetate and lead citrate, and examined with a Hitachi H-7650/transmission electron microscope at 60 kV located at the College of Arts and Sciences Microscopy, Texas Tech University (TTU) and stained for 5 min in lead citrate. They were rinsed and poststained for 30 min in uranyl acetate and then were rinsed again and dried. Electron microscopy was performed at 60 kV on a Hitachi H-7650/transmission electron microscope at 60 kV located at the College of Arts and Sciences Microscopy, TTU equipped with a CCD, and images were collected at magnifications of ×1000–37,000. Then, the mitochondrial number and mitochondrial length were counted in ImageJ for all groups of cells, and statistical significance was determined using a one-way analysis of variance (ANOVA).

### 2.10. Statistical Analysis

The significance of differences among groups was evaluated with one-way ANOVA followed by Tukey’s post hoc test. The significance of differences among groups was calculated with an unpaired, two tailed Student’s *t*-test. Differences were considered statistically significant at *p* < 0.05 (Graph Prism Pad version 6).

## 3. Results

### 3.1. Immunoblotting Analysis of Rlip Protein

**Figure 2:**
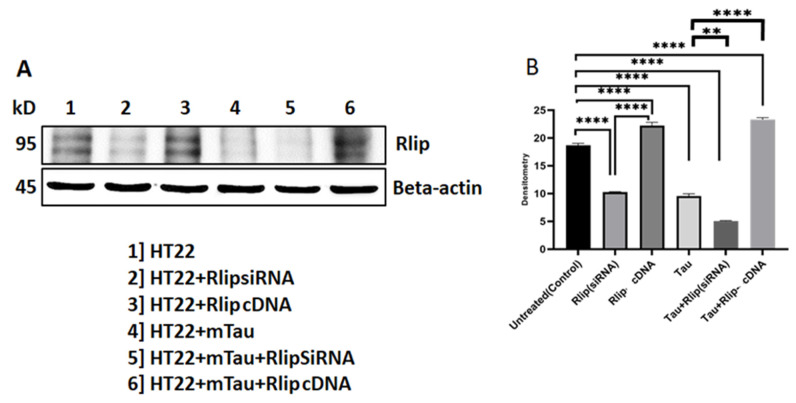
Immunoblotting analysis of Rlip protein. (**A**) Representative immunoblots for control HT22 cells and mTau-HT22 cells with or without Rlip overexpression and silencing. (**B**) Represents quantitative densitometry analysis of Rlip protein. Rlip level was significantly decreased in Mutant Tau when compared with the control. Increased Rlip level was observed in Rlip-cDNA with control cells. Decreased Rlip level was observed in Tau+Rlip (siRNA) when compared with control HT22 cells. However, a significantly increased Rlip level was observed in mutant Tau+Rlip-cDNA when compared with the control. (* *p* = 0.05, ** *p* = 0.005, *** *p* = 0.001, and **** *p* = 0.0001).

As shown in Figure 2, the Rlip level was significantly decreased in mTauHT22 cells when compared with the control HT22 cells (*p* = 0.0001). A decreased Rlip level was observed in mTau + Rlip (siRNA) HT22 cells when compared with the control HT22 cells (*p* = 0.0001). An increased Rlip level was observed in Rlip-cDNA-transfected HT22 cells compared with the control cells (*p* = 0.0002). The Rlip level increased in mTau + Rlip-cDNA-transfected cells when compared with the control cells (*p* = 0.0002). The Rlip level decreased in mTau-HT22 cells+ Rlip silencing (*p* = 0.0003) and significantly increased in mTau HT22 + Rlip overexpression (*p* = 0.0002) as compared to the HT22+mTau cells. Figure 2A Representative immunoblots for control HT22 cells and mTau-HT22 cells with or without Rlip overexpression and silencing. Figure 2B Represents a quantitative densitometry analysis of mitophagy proteins.

### 3.2. Cell Survival Assays in HT22 Cells and HT22 Cells Transfected with Mutant Tau cDNA and Rlip Overexpression and Silencing

**Figure 3:**
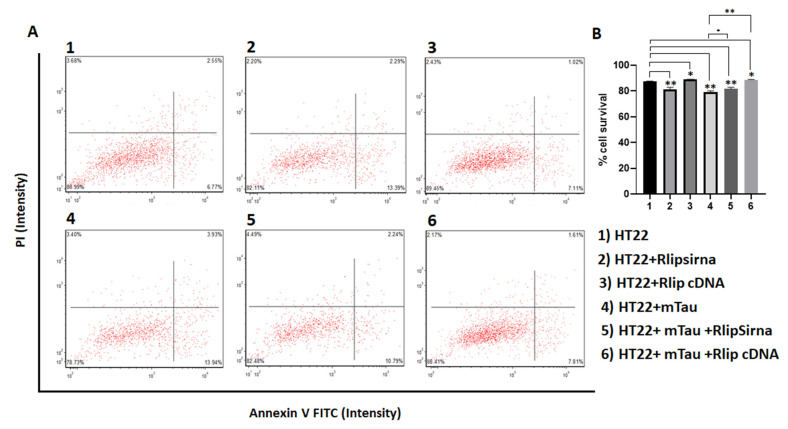
Cell survival assays in HT22 cells and HT22 cells transfected with mutant Tau cDNA and Rlip overexpression and silencing. (**A**) Cell survival was significantly decreased in mutant Tau cells relative to control HT22 cells. (**B**) Quantification of cell survival in each group. There was increased cell survival in Rlip-cDNA with control cells. However, cell survival was increased in Rlip-overexpressed mutant Tau cells relative to control HT22 cells. (* *p* = 0.05, ** *p* = 0.005, *** *p* = 0.001, and **** *p* = 0.0001).

As shown in Figure 3, cell survival was significantly decreased in the RNA-silenced HT22 cells (*p* = 0.001), in the mTau-HT22 cells (*p* = 0.003), and a combination of RNA-silenced+mTau HT22 cells (*p* = 0.002) compared to the HT22 cells. On the other hand, cell survival was significantly increased in the Rlip-overexpressed (HT22 + Rlip cDNA) cells (*p* = 0.01) and in mTau + Rlip-overexpressed HT22 cells (*p* = 0.01) compared to the control HT22 cells. Cell survival also increased in the mTau+Rlip-overexpressed HT22 cells (*p* = 0.001) compared to mTau-HT22 cells. These observations indicate that mTau and RNA-silenced Rlip reduce cell survival.

### 3.3. Mitochondrial Respiration

Basal respiration is the energetic demand of cells under basal conditions. The oxygen consumption of basal respiration is used to achieve ATP synthesis and results in mitochondrial proton leak. ATP-linked respiration is reproduced by the decrease in OCR after the injection of the ATP synthase inhibitor oligomycin (part of basal respiration). The proton leak is the remaining basal respiration which is not united to ATP synthesis after oligomycin injection, and it can lead to mitochondrial damage. Maximal respiration signifies the maximum capacity that the electron respiratory chain can attain. The maximal oxygen consumption rate is measured by injecting the uncoupler FCCP. The difference between maximal and basal respiration is called spare respiration. It imitates the ability of the cells to respond to the changes in energetic demand and shows the fitness of the cells. The other type of respiration measured after the injection of rotenone and antimycin A is called non-mitochondrial respiration, which is the oxygen consumption due to cellular enzymes other than mitochondria.

**Figure 4:**
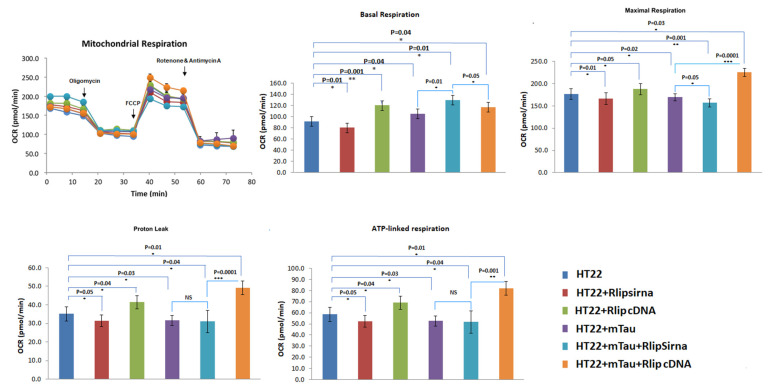
Mitochondrial respiration using Rlip overexpression and silencing in HT22 cells and mutant TauHT22 cells. Kinetic OCR (oxygen consumption rate) response of HT22 cells and mTau HT22 cells transfected with Rlip overexpression/silencing to oligomycin (2 µM) to determine ATP-coupled respiration, FCCP (1 µM) to determine maximal respiration, and rotenone and antimycin A (0.5 µM) to define spare respiratory capacity. Seahorse analyzer records OCR values versus time before and after the injections of oligomycin, FCCP, and rotenone/antimycin A. With these OCR readings, the Mito Stress Test kit explains multiple vital parameters of mitochondrial function including basal respiration (BR), ATP-linked respiration (ATP), proton leak (PL), and maximal respiration (MaxR). To determine the effects of Rlip overexpression and Rlip silencing on mt respiration, we assessed the maximal oxygen consumption rate (OCR) in mTauHT22 cells transfected with Rlip cDNA and RLIP siRNA. Maximal OCR decreased relative to control HT22 cells in mutant Tau cells. Maximal OCR was increased in Rlip-cDNA with control cells. However, maximal OCR was increased in Rlip-overexpressed mutant Tau cells relative to control HT22 cells. (* *p* = 0.05, ** *p* = 0.005, *** *p* = 0.001).

As shown in Figure 4, the maximal OCR was decreased in the mTau-HT22 cells (*p* = 0.02), in the RNA-silenced Rlip-HT22 cells (*p* = 0.01), and in the mTau + RNA-silenced Rlip HT22 cells (*p* = 0.001) relative to the control HT22 cells. An increased maximal OCR was observed in Rlip-cDNA-transfected HT22 cells (*p* = 0.05) and in Rlip-overexpressed mTau-HT22 (*p* = 0.03) compared to the control HT22 cells (Figure 4). A similar pattern was found in ATP-linked respiration and proton leaks. The maximal respiration decreased in mTau-HT22 cells + Rlip silencing (*p* = 0.05) and increased in mTau HT22 + Rlip overexpression (*p* = 0.0001) when compared with HT22 + mTau cells.

### 3.4. Mitochondrial Functional Analysis in HT22 Cells

**Figure 5:**
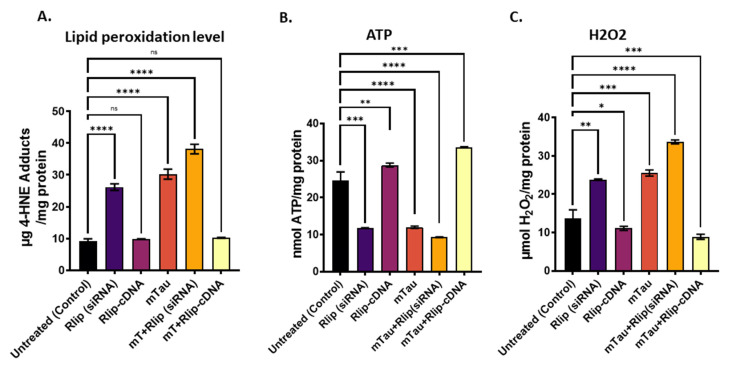
Mitochondrial functional analysis in HT22 cells. (**A**) Lipid peroxidation as measured by 4HNE adducts (**B**) ATP and (**C**) H_2_O_2_ in total cell lysates. Significantly increased production of 4HNE and H_2_O_2_ was found in Tau-transfected cells, which was reduced after Rlip-cDNA cotransfection and untreated controls. ATP production was significantly decreased in Tau-transfected cells compared to Tau and Rlip-cDNA cotransfection. All results are reported as mean ± SD. (* *p* = 0.05, ** *p* = 0.005, *** *p* = 0.001, and **** *p =* 0.0001). (ns-Non-significant).

Figure 5A Lipid peroxidation as measured by 4HNE adducts, Figure 5B ATP and Figure 5C H_2_O_2_ in total cell lysates. Significantly increased production of 4HNE (lipid peroxidation) was found in mTau-transfected HT22 cells (*p* = 0.00001), RNA silencing Rlip HT22 cells (*p* = 0.00001), and mTau + RNA-silenced Rlip HT22 cells (*p* = 0.00001) compared to HT22 cells (Figure 4). On the other hand, 4HNE levels were reduced in mTau-HT22 cells + Rlip-cDNA-transfected cells relative to mTau-HT22 cells (Figure 5A).

ATP production is significantly decreased in mTau cells (*p* = 0.00001), in RNA silenced HT22 cells (*p* = 0.0001) and in mTau + RNA silenced HT22 cells (*p* = 0.00001) compared to control HT22 cells (Figure 5B). As expected, ATP production was increased in Rlip cDNA overexpressed HT22 cells and in in Rlip cDNA overexpressed+mTauHT22 cells (*p* = 0.0001) compared to control HT22 cells.

As shown in Figure 5C, H_2_O_2_ levels were increased in RNA-silenced Rlip HT22 cells (*p* = 0.001) and mTau-transfected HT22 cells (*p* = 0.0001) compared to HT22 cells. On the other hand, H_2_O_2_ levels were reduced in Rlip-cDNA-transfected HT22 cells (*p* = 0.01) and in mTau-HT22+Rlip cDNA cells (*p* = 0.0001) compared to HT22 cells.

### 3.5. Immunoblotting Analysis of Mitophagy Proteins

**Figure 6:**
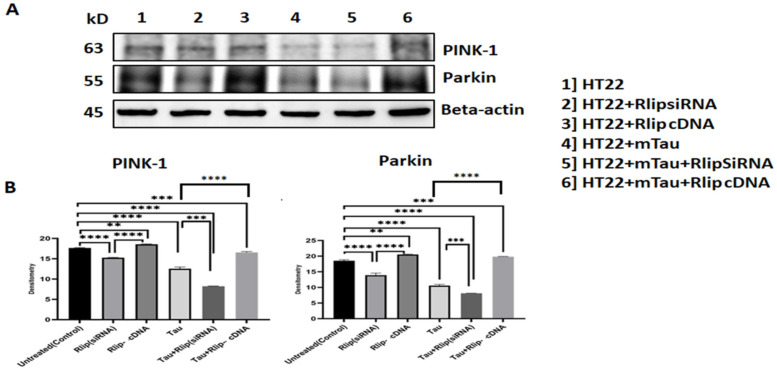
Immunoblotting analysis of mitophagy proteins. (**A**) Representative immunoblots for control HT22 cells and mTau-HT22 cells with or without Rlip overexpression and silencing. (**B**) Represents quantitative densitometry analysis of mitophagy proteins. Pink 1 and Parkin levels were significantly decreased in Mutant Tau when compared with the control. Increased Pink 1 and Parkin levels were observed in Rlip-cDNA with control cells. Decreased Pink 1 and Parkin levels were observed in Tau + Rlip (siRNA) when compared with control HT22 cells. However, significantly increased Pink and Parkin levels were observed in mutant Tau + Rlip-cDNA when compared with the control. (** *p* = 0.005, *** *p* = 0.001, and **** *p =* 0.0001).

As shown in Figure 6, PINK 1 and Parkin levels were significantly decreased in the mTauHT22 cells when compared with the control HT22 cells (*p* = 0.0001, *p* = 0.0002). Decreased PINK 1 and Parkin levels were observed in the mTau+Rlip (siRNA) HT22 cells when compared with the control HT22 cells (*p* = 0.0001, *p* = 0.0001). Increased PINK 1 and Parkin levels were observed in the Rlip-cDNA-transfected HT22 cells compared with the control cells (*p* = 0.002). PINK and Parkin levels increased in the mTau + Rlip-cDNA-transfected cells when compared with the control cells (*p* = 0.0001, *p* = 0.0002). PINK 1 and Parkin levels decreased in the mTau-HT22 cells+ Rlip silencing (*p* = 0.0003) (*p* = 0.0001) and significantly increased in mTau HT22 + Rlip overexpression (*p* = 0.0002) (*p* = 0.0001) as compared to the HT22 + mTau cells. Figure 6A Representative immunoblots for control HT22 cells and mTau-HT22 cells with or without Rlip overexpression and silencing. Figure 6B Represents a quantitative densitometry analysis of mitophagy proteins.

### 3.6. Immunofluorescence Analysis of Mitophagy and Synaptic Proteins

**Figure 7:**
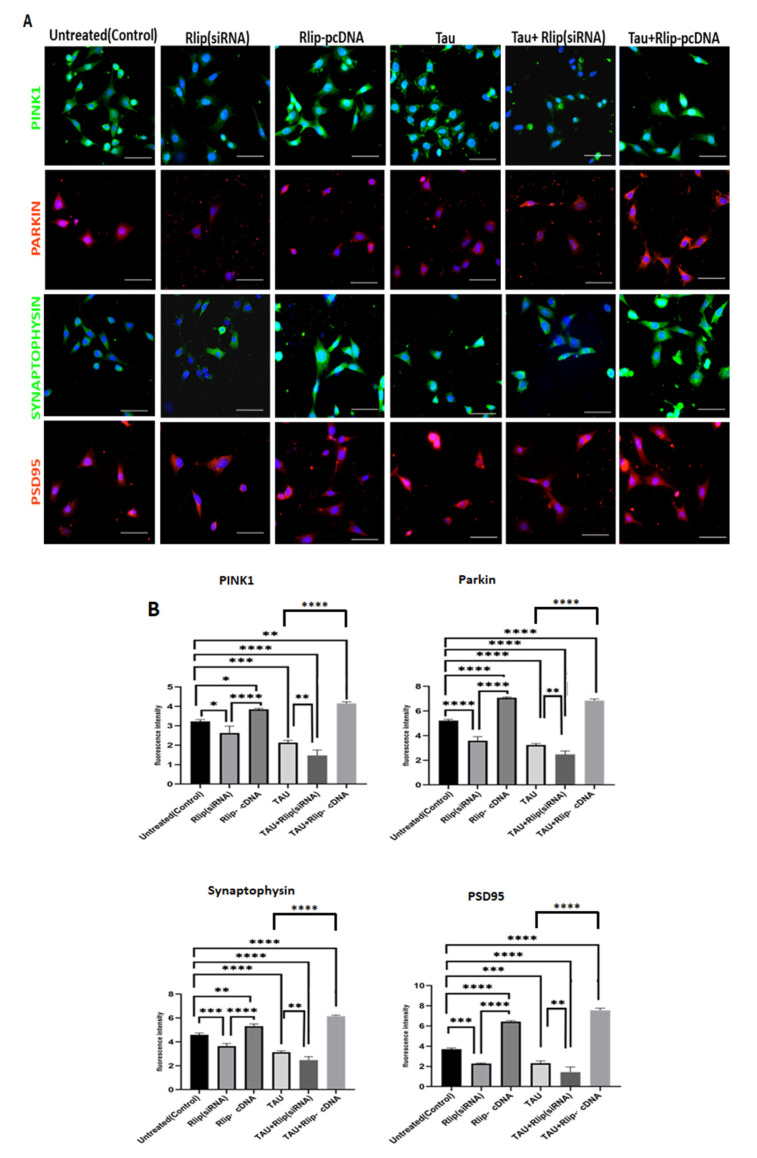
(**A**) Immunofluorescence analysis of mitophagy (PINK1 and Parkin) and synaptic proteins (Synaptophysin and PSD95). (**B**) PINK1 levels were significantly decreased in mTau cells when compared with the control. Increased PINK1 levels were observed in Rlip-cDNA with control cells. PARKIN levels were significantly decreased in mTau-HT22 cells when compared with the control. Synaptophysin levels were significantly decreased in mTau-HT22 cells when compared with the control. Increased Synaptophysin levels were observed in Rlip-cDNA with control cells. PSD95 levels were significantly decreased in mTau-HT22 cells when compared with the control. (* *p* = 0.05, ** *p* = 0.005, *** *p* = 0.001, and **** *p* = 0.0001).

Significantly decreased PINK1 levels were found in the mTauHT22 cells when compared with the control HT22 cells (*p* = 0.0002) (Figure 7A,B). Decreased PINK1 was observed in mTau + Rlip (siRNA) when compared with the control HT22 cells (*p* = 0.0001). However, a significantly increased PINK1 level was observed in mTau+Rlip-cDNA-transfected cells when compared with the control cells (*p* = 0.005). Increased PINK1 levels were observed in the Rlip-cDNA-transfected cells compared to the control cells (*p* = 0.05). The PINK 1 level was decreased in mTau + Rlip (siRNA) (*p* = 0.002) as compared to the mTau HT22-cells. An increased PINK1 level was observed in the mTau + Rlip-cDNA-transfected cells (*p* = 0.0001) when compared to the mTau HT22 cells.

PARKIN levels were significantly decreased in the mTau-HT22 cells when compared with the control (*p* = 0.0001) (Figure 7). Decreased PARKIN was observed in the mTau + Rlip (siRNA) cells compared with the control HT22 cells (*p* = 0.0002). However, significantly increased PARKIN levels were observed in the Rlip-cDNA-transfected HT22 cells compared with the control cells (*p* = 0.0002). Increased PARKIN was observed in the mutant Tau + Rlip-cDNA-transfected HT22 cells when compared with the control (*p* = 0.0003). The Parkin level was decreased in mTau + Rlip (siRNA) (*p* = 0.003) as compared to the mTau HT22-cells. An increased Parkin level was observed in the mTau + Rlip-cDNA-transfected cells (*p* = 0.0001) when compared to the mTau HT22 cells. (Scale bar 10 μM.)

Significantly decreased synaptophysin levels were observed in the mTau-HT22 cells when compared with the control cells (*p* = 0.0001). Decreased Synaptophysin was observed in mTau + Rlip (siRNA) when compared with the control HT22 cells (*p* = 0.0003). On the other hand, significantly increased synaptophysin was observed in the mTau + Rlip-cDNA-transfected cells when compared with the control cells (*p* = 0.0004). Increased synaptophysin levels were observed in the Rlip-cDNA-transfected cells when compared with the control cells (*p* = 0.003). The Synaptophysin level was decreased in mTau + Rlip (siRNA) (*p* = 0.003) as compared to the mTau HT22-cells. An increased Synaptophysin level was observed in the mTau+Rlip-cDNA-transfected cells (*p* = 0.0001) when compared to the mTau HT22 cells.

Significantly decreased PSD95 levels were observed in the mTau-HT22 cells when compared with the control cells (*p* = 0.0001). Decreased PSD95 levels were observed in mTau + Rlip (siRNA) when compared with the control HT22 cells (*p* = 0.0003). Significantly increased PSD95 was observed in mTau + Rlip-cDNA when compared with the control cells (*p* = 0.0001). Increased PSD95 levels were observed in Rlip-cDNA with control cells (*p* = 0.0002). The PSD95 level decreased in mTau + Rlip (siRNA) (*p* = 0.002) as compared to the mTau HT22-cells. An increased PSD95 level was observed in the mTau+Rlip-cDNA-transfected cells (*p* = 0.0001) when compared to mTau HT22 cells.

### 3.7. Immunoblotting Analysis of Synaptic Proteins

**Figure 8:**
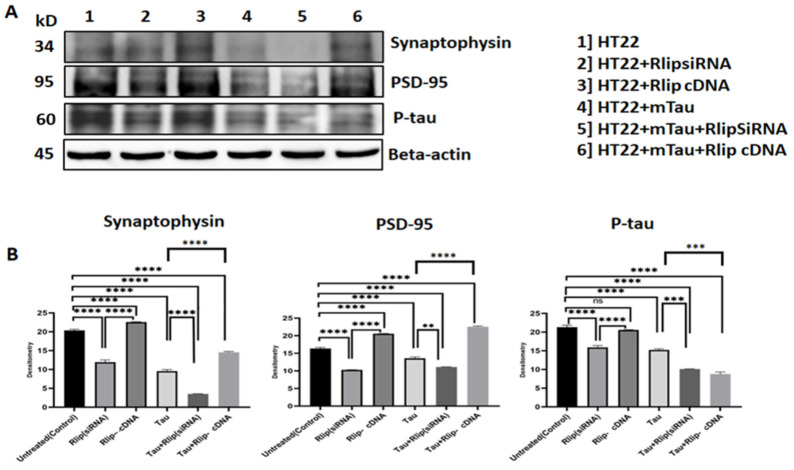
Immunoblotting analysis of synaptic proteins. (**A**) Representative immunoblots for control HT22 cells and mTau-HT22 cells with or without Rlip overexpression and silencing. (**B**) represents quantitative densitometry analysis of synaptic proteins. Synaptophysin and PSD95 levels were significantly decreased in Mutant Tau when compared with the control. Increased Synaptophysin and PSD95 levels were observed in Rlip-cDNA with control cells. Decreased Synaptophysin and PSD95 were observed in Tau + Rlip (siRNA) when compared with control HT22 cells. However, significantly increased Synaptophysin and PSD95 were observed in mutant Tau + Rlip-cDNA when compared with the control. (** *p* = 0.005, *** *p* = 0.001, and **** *p* = 0.0001, ns = not significant).

As shown in Figure 8, the synaptophysin and PSD95 levels significantly decreased in the mTau-HT22 cells compared with the control HT22 cells (*p* = 0.0003, *p* = 0.05). Decreased synaptophysin and PSD95 were observed in the RNA-silenced HT22 cells (*p* = 0.00001, *p* = 0.00001) compared to the control HT22 cells, as well as mTau + Rlip (siRNA) when compared with the control HT22 cells (*p* = 0.0002, *p* = 0.008) (Figure 8). On the other hand, synaptic proteins, synaptophysin, and PSD95 levels were increased in the Rlip-cDNA-transfected HT22 cells relative to the control cells (*p* = 0.001, *p* = 0.003). Significantly increased synaptophysin and PSD95 were observed in the mTau + Rlip-cDNA-transfected HT22 cells when compared with the control cells. Figure 8A shows representative immunoblots for the control HT22 cells and mTau-HT22 cells with or without Rlip overexpression and silencing (Figure 8). Figure 8B represents a quantitative densitometry analysis of synaptic proteins. The levels of synaptic proteins Synaptophysin and PSD95 were decreased in mTau + Rlip (siRNA) (*p* = 0.0001) (*p* = 0.002) as compared to the mTau HT22 cells. Increased Synaptophysin and PSD95 levels were observed in the mTau + Rlip-cDNA-transfected cells (*p* = 0.0001) (*p* = 0.0001) when compared to the mTau HT22 cells. Significantly decreased *p*-tau levels were found in the Rlip-overexpressed mTau-HT22 cells. P-tau levels were significantly increased in the mTau-HT22 cells when compared with the control HT22 cells (*p* = 0.0001). Decreased p-tau levels were observed in the Rlip-overexpressed HT22 cells when compared with the control HT22 cells. Significantly increased p-tau levels were observed in mTau + Rlip (siRNA) when compared with the mTau-HT22 cells (*p* = 0.0005) and also decreased p-tau levels were observed in the mTau-HT22 cells with Rlip overexpression compared with the control HT22 cells (*p* = 0.0003). (*p* < 0.0001, *p* < 0.0001). (* *p* = 0.05, ** *p* = 0.005, *** *p* = 0.001, and **** *p =* 0.0001).

### 3.8. Immunofluorescence Analysis and Colocalization of RLIP and Mutant Tau Proteins

**Figure 9:**
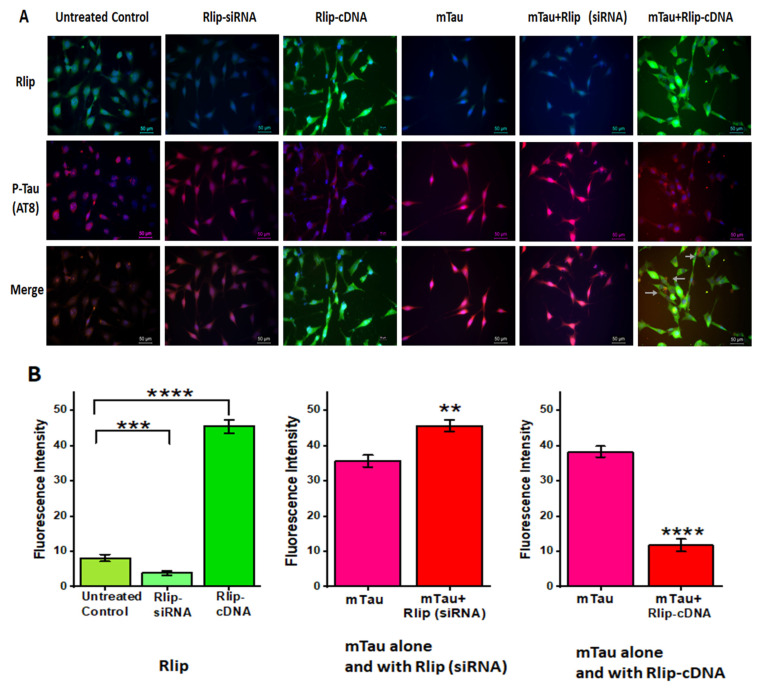
Immunofluorescence analysis and colocalization of RLIP and mutant Tau proteins. Immunofluorescence analysis of Rlip and mTau in control HT22 cells and mTau-HT22 cells with or without Rlip overexpression and silencing. (**A**) representative immunofluorescence images and (**B**) quantitative analysis of mTau and Rlip proteins in HT22 cells and mTau-HT22 cells with or without Rlip overexpression and silencing. (* *p* = 0.05, ** *p* = 0.005, *** *p* = 0.001, and **** *p =* 0.0001).

To determine whether Rlip colocalizes with mTau, we transfected mutant Tau cDNA and Rlip cDNA together in HT22 cells for 24 h in coverslips and performed colocalization studies. As shown in Figure 9, Rlip immunoreactivity was significantly decreased in the RNA-silenced HT22 cells (*p* = 0.001); on the other hand, the immunoreactivity of Rlip was significantly increased (*p* = 0.0001) in the Rlip-overexpressed HT22 cells. Mutant Tau levels were significantly increased in the Rlip-RNA-silenced HT22 cells (*p* = 0.001) relative to the mTau-HT22 cells, and mutant Tau levels were significantly decreased in the Rlip-overexpressed HT22 cells (*p* = 0.0001) compared to the mTau-HT22 cells.

### 3.9. Transmission Electron Microscopy Analysis

**Figure 10:**
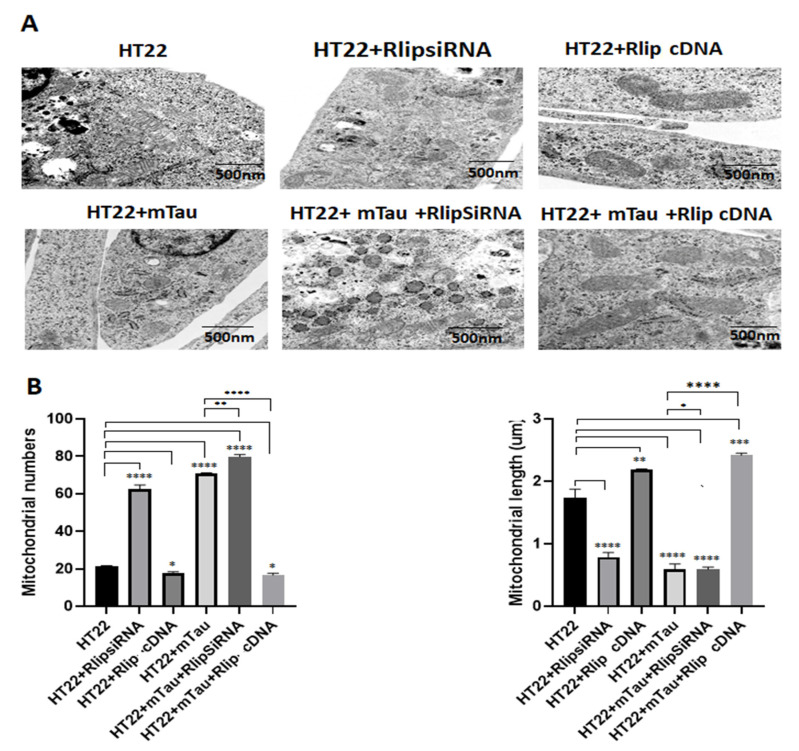
Transmission electron microscopy analysis. Mitochondrial number and length in control HT22 cells and mTau-HT22 cells with or without Rlip overexpression and silencing for 24 h. (**A**) Representative transmission electron microscopy images of mitochondria in the untreated HT22 cells and mutant Tau cDNA HT22 cells transfected with Rlip-cDNA and Rlip siRNA. (**B**) Quantitative analysis of mitochondrial number and length in each of the 6 groups. A significantly increased number of mitochondria was found in HT22 cells transfected with mutant Tau relative to untransfected HT22 cells. Mitochondrial length significantly decreased upon mutant Tau cDNA transfection. Rlip overexpression decreased the mitochondrial number and increased its length in the mTauHT22 cells. (* *p* = 0.05, ** *p* = 0.005, *** *p* = 0.001, and **** *p =* 0.0001).

We performed mitochondrial morphology (mitochondrial number and length) analysis in all six groups of cells in order to determine the impact of Rlip RNA silencing and/or overexpression on the presence of mutant Tau in HT22 cells (Figure 10). Figure 10A shows a representative transmission electron microscopy of mitochondria. Figure 10B shows a quantitative analysis of mitochondrial number and length in each of the six groups.

A significantly increased number of mitochondria was found in the HT22 cells transfected with mutant-Tau-cDNA-transfected cells relative to the untransfected HT22 cells (*p* = 0.0001). Mitochondrial length was significantly decreased in the mutant-Tau-cDNA-transfected cells (*p* = 0.0001). Mutant Tau + Rlip overexpression HT22 cells showed a decreased mitochondrial number (*p* = 0.02) and increased length (*p* = 0.0004) in the mTau-HT22 cells relative to the control cells. The mitochondrial numbers significantly decreased in HT22+mTau Rlip-overexpressed cells (*p* = 0.0001) and the mitochondrial length increased in the HT22+mTau Rlip-overexpressed cells (*p* = 0.001) as compared to the mTau HT22 cells.

## 4. Discussion

The objective of our study is to understand the role of Rlip in the progression of Alzheimer’s disease neurons, particularly AD-affected hippocampal neurons that express mutant Tau (we refer to mTau-HT22 cells). To achieve our objective, we used Rlip-silenced and Rlip-overexpressed strategies in mTau-HT22 cells and studied cell survival, mitochondrial respiration, mitochondrial function (free radicals, lipid peroxidation, and ATP), synaptic and mitophagy proteins, the localization and colocalization of Rlip and mutant Tau proteins, and mitochondrial morphology. We compared the data in every possible way to draw meaningful outcomes.

We found that Rlip protein levels were reduced in mTau, Rlip-silenced HT22 cells and mTau + Rlip-silenced HT22 cells; on the other hand, increased Rlip levels were observed in Rlip-cDNA-transfected HT22 cells compared with control cells. Cell survival was significantly decreased in mTau-HT22 cells and in Rlip-RNA-silenced HT22 cells, and also in combination with mTau-HT22 + Rlip-RNA-silenced HT22 cells. As expected, increased cell survival was observed in Rlip-overexpressed mTau-HT22 cells in all combinations. The oxygen consumption rate was decreased in mTau-HT22 cells and Rlip-RNA-silenced HT22 cells, and was further reduced in mTau+Rlip-RNA-silenced HT22 cells. Mitochondrial function was defective in mTau-HT22 cells and Rlip-RNA-silenced HT22 cells, and further reduced in mTau+Rlip-RNA-silenced HT22 cells; however, cell survival was rescued in Rlip-overexpressed mTau-HT22 cells. Synaptic mitophagy proteins were decreased in mTau-HT22 cells. However, these were increased in mTau+Rlip-overexpressed HT22 cells. An increased number of mitochondria and decreased mitochondrial length were found in mTau-HT22 cells. These were rescued in Rlip-overexpressed mTau-HT22 cells. These observations suggest that Rlip reduction is toxic to AD neurons and this effect is rescued by Rlip overexpression.

As observed in the current study, mutant Tau reduced cell survival, mitochondrial respiration, mitochondrial function, and synaptic and mitophagy proteins, and altered mitochondrial morphology (increased mitochondrial number and reduced mitochondrial length). These observations concur with earlier in vitro [33] and in vivo studies [34]. The current study findings revealed that phosphorylated tau levels were increased in mTau-HT22 cells and also in Rlip-RNA-silenced HT22 cells, indicating that Rlip RNA silencing plays a key role in phosphorylated-tau-induced toxicities, in addition to mTau in HT22 cells. Increasing evidence also suggests that phosphorylated tau is highly toxic to AD, particularly late-onset AD and other tauopathies [33,34,35,36,37]. Recent research also suggests that hyperphosphorylated Tau induces free radical production and lipid peroxidation and reduces antioxidant enzymes (e.g., glutathione peroxidase and catalase), causing an imbalance between the production of free radicals and endogenous antioxidant enzymes, ultimately leading to mitochondrial dysfunction in AD and other tauopathies [34,38,39,40]. Furthermore, mutant tau overexpression in cells showed negative consequences for anterograde mitochondrial transport, directly inhibiting the kinesin-1 and causing a road block on the microtubule [35,36,37]. Our recent Rlip heterozygous knockout (Rlip^+/−^) mice studies revealed increased free radicals and lipid peroxidation and reduced ATP, indicating that reduced Rlip is involved with oxidative stress and mitochondrial dysfunction [28]. Overall, these studies, together with the current study findings, suggest that mutant and/or phosphorylated tau is toxic to AD neurons.

Reduced Rlip is believed to induce oxidative stress and mitochondrial dysfunction (increase free radicals and 4-HNE levels (lipid peroxidation), and inhibit mitochondrial ATP in neurons) [27]. The current study findings of RNA-silenced HT22 cells showed toxic effects in all parameters studied; this effect is even stronger in RNA-silenced+mTau-HT22 cells for all parameters in the current study, particularly for oxidative stress and mitochondrial dysfunction. The reduction in Rlip is not good for AD neurons in many ways, reduces synaptic and mitophagy activities, and reduces mitochondrial ATP, which is critical to maintaining cell homeostasis. The current study observations agree with our recent heterozygous Rlip knockout (Rlip^+/−^) mice findings of oxidative stress/mitochondrial function/dysfunction, synaptic, and mitochondrial morphology. What is the mechanistic input here? Normal Rlip expression is critical to maintaining oxidative stress and mitochondrial function; it is possible that increased phosphorylated tau suppresses Rlip levels in disease progression.

In the current study, Rlip overexpression showed increased cell survival, mitochondrial respiration and function, enhanced synaptic/mitochondrial proteins and maintained mitochondrial dynamics, reduced mitochondrial number, and increased mitochondrial length in the hippocampal neurons. Mechanistically, the overexpression of Rlip reduces oxidative stress and mitochondrial function by scavenging free radicals and reducing oxidative stress. Our study is the first to report that Rlip overexpression is beneficial to hippocampal cells that express mutant Tau. Furthermore, these in vitro study observations warrant further investigations using overexpressed Rlip in mice or Rlip transgenic mice. To understand the impact of Rlip depletion and/or overexpression, it is critical to cross these mice with mice that express Tau mutations. Mutant-Tau-expressed and Rlip-RNA-silenced cells showed highly significant toxicity to the hippocampal neurons in almost all parameters investigated in the current study—cell survival, mitochondrial respiration, synaptic and mitophagy defects, and mitochondrial damage and mitochondrial function. On the other hand, as observed in the current in vitro study, Rlip overexpression rescued and/or reduced mutant Tau toxicities in almost aspects studied. Some mechanistic aspects of how mutant and/or phosphorylated tau cause toxicity to AD cells are given here: (1) mutant tau interaction with mitochondrial protein Drp1 forms a complex that disrupts the axonal transport of mitochondria [41], (2) mutant tau overexpression in cells directly inhibits kinesin-1, causing a road block on the microtubule [42,43], (3) N-terminal Tau enters the mitochondria and induces free radicals, increases lipid peroxidation, inhibits cytochrome c oxidase, and reduces mitochondrial ATP, and mutant Tau reduces mitophagy and synaptic proteins disrupt mitophagy and synaptic activities [34,42,44,45,46]. (4) Furthermore, it is possible that normal or wild-type Rlip physically interacts with normal tau and maintains cell homeostasis (oxidative stress, a balance between free radicals and antioxidant enzymes, and mitochondrial function) that is disrupted with age and mutant-tau-dependent increased hyperphosphorylated tau. In addition, (5) Rlip is a surface protein that maintains the dynamic balance between exocytosis-mediated surface delivery and endocytosis-dependent retrieval from the cell surface. Imbalances in surface protein levels perturb surface protein homeostasis in AD and other tauopathies [47]. (6) It is possible that increased phosphorylated tau and/or amyloid beta reduces Rlip levels and its surface protein activities in the disease process, particularly vesicle-bound small guanosine triphosphatases of Rlip that promote exocytosis through interacting with the exocyst complex. (7) it is also possible that Rlip plays a role in mitochondrial dynamics (fission–fusion balance), mitochondrial biogenesis, and, most importantly, mitophagy activities in aging, AD, and other tauopathies/neurological diseases. Detailed investigations are urgently needed to unravel these aspects. These possibilities can be addressed using in vivo studies by crossing Rlip^+/−^ mice and/or Rlip transgenic mice with transgenic Tau mice. These genetic crossing studies will allow us to examine (1) time-course cellular and pathological changes, (2) gender-based cellular changes, (3) region-specific changes in the brain and also impact on the electron transport chain, ATPase activity and mitochondrial enzymatic activities in double mutant mice lines—Rlip^+/−^ X Tau Tg; Rlip Tg X Tau Tg).

In summary, we cautiously conclude Rlip is a promising new target for aging, AD, and other tauopathies/neurological diseases that have shown the relevance of oxidative stress and mitochondrial dysfunction in the disease process. The findings from this study strongly suggest that Rlip deficiency causes oxidative stress/mitochondrial dysfunction, reduced cell survival and mitochondrial respiration, reduced synaptic and mitophagy activities, and defective mitochondrial morphology. Rlip overexpression reverses these defects. As mentioned above, a thorough investigation of Rlip protein is urgently needed in relation to gender, sex, age, and regional specificity, not only in mouse models but also in postmortem brains with different stages of AD progression and other tauopathies. Our current in vitro study is the first to describe the role of Rlip in the presence and absence of mutant Tau.

## Figures and Tables

**Table 1:** Summary of antibody dilutions and conditions used in the immunoblotting analysis of Rlip, synaptic, and mitophagy proteins in mTau HT22 cells transfected with Rlip overexpression and Rlip silencing.

Marker Primary Antibody—Species and Dilution	Company, City, and State of Purchase	Secondary Antibody, Dilution	Company, City, and State of Purchase
RalBP1/Rlip Rabbit (polyclonal) # 3630S 1:1000	Cell Signaling Technology Danvers, MA, USA	Donkey anti-rabbit HRP 1:10,000	GE Healthcare Amersham, Piscataway, NJ, USA
Phospho-Tau (Ser202, Thr205) (AT8) Mouse (monoclonal) 1:500 #MN1020	Invitrogen (ThermoFisher Sci.) Waltham, MA, USA	Sheep anti-mouse HRP 1:10,000	GE Healthcare Amersham, Piscataway, NJ, USA
SYN Rabbit monoclonal 1:400 #Ab32127	Abcam, Cambridge, MA, USA	Donkey anti-rabbit HRP 1:10,000	GE Healthcare Amersham, Piscataway, NJ, USA
PSD-95 Mouse Monoclonal Antibody 1:500 # MA1-046	Invitrogen, Waltham, MA, USA	Sheep anti-mouse HRP 1:10,000	GE Healthcare Amersham, Piscataway, NJ, USA
PINK1 Rabbit polyclonal 1:500 #BC100-494	Novus Biological, Littleton, CO, USA	Donkey anti-rabbit HRP 1:10,000	GE Healthcare Amersham, Piscataway, NJ, USA
Parkin Mouse polyclonal 1:500 #NBP2-29838	Novus Biological, Littleton, CO, USA	Sheep anti-mouse HRP 1:10,000	GE Healthcare Amersham, Piscataway, NJ, USA

**Table 2:** Primary and secondary antibodies used for IF (immunofluorescence).

Antibody	Species	Dilution Used	Supplier	Catalog Number
RalBP1	Mouse (monoclonal)	1:100	Origene Technology	TA500964
SYN	Rabbit monoclonal	1:100	Abcam, Cambridge, MA, USA	Ab32127
PSD-95	Mouse Monoclonal Antibody	1:100	Invitrogen, Waltham, MA, USA	MA1-046
PINK1	Rabbit polyclonal	1:100	Novus Biological, Littleton, CO, USA	BC100-494
Parkin	Mouse polyclonal	1:100	Novus Biological, Littleton, CO, USA	NBP2-29838
Phospho-Tau (Ser202, Thr205) (AT8)	Mouse (monoclonal)	1:100	Invitrogen (ThermoFisher Sci.)	MN1020
Anti-mouse IgG AlexaFluor488	Goat	1:100	Invitrogen (ThermoFisher Sci.)	A-11001
Anti-rabbit (H+L), (Texas-red-conjugated)	Goat	1:100	Novus Biologicals	NB120-6719

## Data Availability

Data generated and analyzed in relation to the studies reported here are provided in this manuscript.
