# Correction: Reddy et al. Rlip Reduction Induces Oxidative Stress and Mitochondrial Dysfunction in Mutant Tau-Expressed Immortalized Hippocampal Neurons: Mechanistic Insights. *Cells* 2023, *12*, 1646

**DOI:** 10.3390/cells13020145

**Published:** 2024-01-12

**Authors:** P. Hemachandra Reddy, Sudhir Kshirsagar, Chhanda Bose, Jangampalli Adi Pradeepkiran, Ashly Hindle, Sharda P. Singh, Arubala P. Reddy, Javaria Baig

**Affiliations:** 1 Department of Internal Medicine, Texas Tech University Health Sciences Center, Lubbock, TX 79430, USA; sudhir.kshirsagar@ttuhsc.edu (S.K.); chhanda.bose@ttuhsc.edu (C.B.); pradeep.jangampalli@ttuhsc.edu (J.A.P.); ashly.hindle@ttuhsc.edu (A.H.); sharda.singh@ttuhsc.edu (S.P.S.); jbaig@ttuhsc.edu (J.B.); 2 Nutritional Sciences Department, College of Human Sciences, Texas Tech University, Lubbock, TX 79409, USA; arubala.reddy@ttu.edu; 3 Neurology, Departments of School of Medicine, Texas Tech University Health Sciences Center, Lubbock, TX 79430, USA; 4 Public Health Department of Graduate School of Biomedical Sciences, Texas Tech University Health Sciences Center, Lubbock, TX 79430, USA; 5 Department of Speech, Language and Hearing Sciences, School Health Professions, Texas Tech University Health Sciences Center, Lubbock, TX 79430, USA; 6 Department of Pharmacology and Neuroscience, Texas Tech University Health Sciences Center, Lubbock, TX 79430, USA

The authors wish to make the following changes to their paper [1]. 

We accidentally uploaded unchecked versions of Figures 6 and 8 during the revision process. Upon closer examination, we found that the PINK1 (in Figure 6) and synaptophysin (in Figure 8) blots are similar in the published article. We replaced the PINK1 and synaptophysin blots in Figures 6 and 8. We sincerely apologize for our oversight. The corrected Figure 6 and Figure 8 appear below. The authors state that the scientific conclusions are unaffected. This correction was approved by the Academic Editor. The original publication has also been updated.

## Figures and Tables

**Figure 6:**
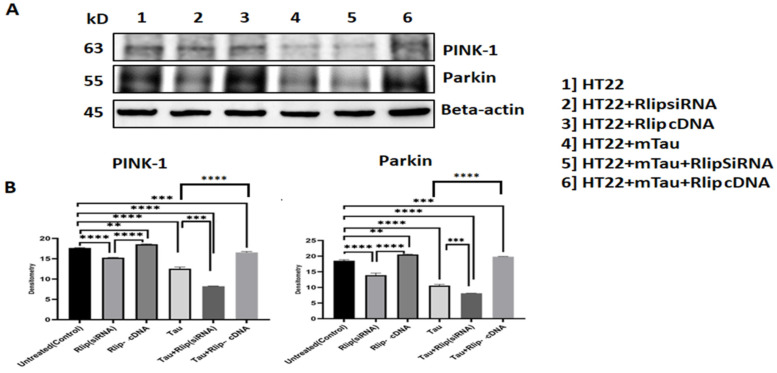
Immunoblotting analysis of mitophagy proteins. (**A**) Representative immunoblots for control HT22 cells and mTau-HT22 cells with or without Rlip overexpression and silencing. (**B**) Represents quantitative densitometry analysis of mitophagy proteins. Pink 1 and Parkin levels were significantly decreased in Mutant Tau when compared with the control. Increased Pink 1 and Parkin levels were observed in Rlip-cDNA with control cells. Decreased Pink 1 and Parkin levels were observed in Tau + Rlip (siRNA) when compared with control HT22 cells. However, significantly increased Pink and Parkin levels were observed in mutant Tau + Rlip-cDNA when compared with the control. (** *p* = 0.005, *** *p* = 0.001, and **** *p =* 0.0001).

**Figure 8:**
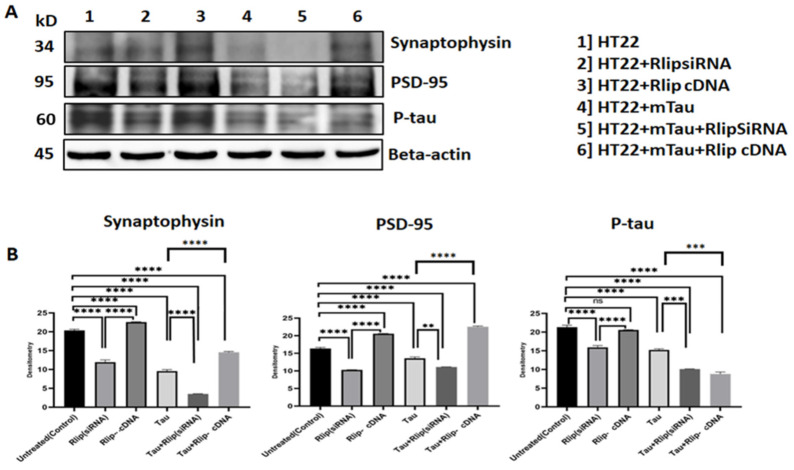
Immunoblotting analysis of synaptic proteins. (**A**) Representative immunoblots for control HT22 cells and mTau-HT22 cells with or without Rlip overexpression and silencing. (**B**) represents quantitative densitometry analysis of synaptic proteins. Synaptophysin and PSD95 levels were significantly decreased in Mutant Tau when compared with the control. Increased Synaptophysin and PSD95 levels were observed in Rlip-cDNA with control cells. Decreased Synaptophysin and PSD95 were observed in Tau + Rlip (siRNA) when compared with control HT22 cells. However, significantly increased Synaptophysin and PSD95 were observed in mutant Tau + Rlip-cDNA when compared with the control. (** *p* = 0.005, *** *p* = 0.001, and **** *p* = 0.0001, ns = not significant).
